# PIK3C3 regulates the expansion of liver CSCs and PIK3C3 inhibition counteracts liver cancer stem cell activity induced by PI3K inhibitor

**DOI:** 10.1038/s41419-020-2631-9

**Published:** 2020-06-08

**Authors:** Fengchao Liu, Xiaoling Wu, Yanzhi Qian, Xin Jiang, Yiying Wang, Jian Gao

**Affiliations:** 1 Department of Gastroenterology, Second Affiliated Hospital, Chongqing Medical University, Chongqing, China; 2 Liver Disease Center, The Affiliated Hospital of Qingdao University, Qingdao, China

**Keywords:** Diagnostic markers, Cancer stem cells

## Abstract

The existence of cancer stem cells (CSCs) accounts for hepatocellular carcinoma (HCC) treatment resistance, relapse, and metastasis. Although the elimination of cancer stem cells is crucial for cancer treatment, strategies for their elimination are limited. Here, we report that a remarkable increase in PIK3C3 was detected in HCC tissues and liver CSCs. Upregulated PIK3C3 facilitated liver CSC expansion in HCC cells; RNA interference-mediated silencing of PIK3C3 had an opposite effect. Furthermore, PIK3C3 inhibition by inhibitors effectively eliminated liver CSCs and inhibited the growth of tumors in vivo. The phosphoinositide 3-kinase (PI3K) pathway is considered an important hallmark of cancer. One of our recent studies found that prolonged inhibition by inhibitors of class I PI3K induces liver CSCs expansion. To our surprise, PIK3C3 inhibition blocked the expansion of CSCs induced by PI3K inhibitor; moreover, treatment with the combination of PIK3C3 inhibitor and PI3K inhibitor in maximal suppresses the expansion of liver CSCs of tumors in mice. Mechanistically, inhibition of PIK3C3 inhibit the activation of SGK3, a CSCs promoter, induced by PI3K inhibitor. We also show that PIK3C3 inhibitor suppresses liver CSCs by activation of the AMP-activated kinase (AMPK). Although PIK3C3 plays a critical role in autophagy, we find that PIK3C3 regulates liver CSCs independent of the autophagy process. These findings uncover the effective suppression of liver CSCs by targeting PIK3C3, and targeting PIK3C3 in combination with PI3K inhibitor inhibits the expansion of liver CSCs efficiently, which is an attractive therapeutic regimen for the treatment of HCC.

## Introduction

Hepatocellular carcinoma (HCC) is the second most common cause of cancer-related death worldwide and has an incidence of approximately 850,000 new cases per year^1,2^. Although chemotherapy, including molecular targeting therapy, surgical resection, and liver transplantation have made significant progress, HCC recurrence occurs frequently and metastasizes, leading to the poor overall survival of HCC^3,4^. There is accumulating evidence that cancer recurrence, metastasis, and treatment resistance in tumors is due to the presence of CSCs^5,6^. In HCC, A growing body of research has demonstrated the existence of CSCs, and several CSC markers have been identified, including CD133, CD90, CD44, EpCAM, OV6, CD47, CD13, CD24, ICAM-1, Lgr5, and keratin19^7^. Although the elimination of cancer stem cells is crucial for cancer treatment, strategies for their elimination are limited.

The Class III phosphoinositide 3-kinase (PIK3C3), also called vacuolar protein sorting 34 (Vps34), plays important roles in the control of both autophagic and endocytic trafficking systems^8,9^, and these systems are critical in a wide range of cellular processes. Furthermore, the lipid kinase activity of PIK3C3 acts as a major source of phosphatidylinositol-3-phosphate (PtdIns3P) in cells, which functions as a secondary messenger or docking signal for proteins containing PtdIns3P-binding domains, such as FYVE or PX^10^. Although accumulating evidence indicates that Vps34 may play a critical role in the progression of cancers, including colon cancer^11^ and breast cancer^12,13^, the role and mechanism of PIK3C3 in CSCs are unknown.

AMP-activated kinase (AMPK) acts as an energetic biosensor and regulator in cells that control cellular energy balance. ATP levels is maintained by the activation of AMPK in response to energy stress caused by mitochondrial dysfunction, hypoxia, or shortage of essential metabolic fuels^14^. Accumulating evidence has reported that AMPK exerts therapeutic effects in tumors^15,16^. Consistently, recent studies also indicate that AMPK activation inhibits the self-renewal of CSCs^17–19^. However, whether AMPK also acts as a suppressor in liver CSCs is unclear. It has been reported that PIK3C3 knockdown, or pharmacological inhibition of PIK3C3, leads to AMPK activation^11,20^. These results support the potential utility of PIK3C3 as a target for CSCs.

The phosphoinositide 3-kinase (PI3K) pathway is an important signaling pathway in malignant process of cancers. Some studies have investigated the therapeutic effect targeting of the PI3K pathway in various cancers, and multiple pharmaceutical inhibitors targeting PI3K signaling have been discovered. Unfortunately, acquired or intrinsic treatment resistance limits the therapeutic efficacy of inhibitors^21,22^. Therefore, elucidating the possible mechanisms underlying the treatment resistance to PI3K inhibitors is required, which may provide a therapeutic schedule for combination therapies or alternative therapies. However, the specific mechanisms are not completely clear. One of our recent studies found that prolonged inhibition by inhibitors of class I PI3K induce liver CSCs expansion^23^, which may be a main reason why HCC cells tolerate the therapy of PI3K inhibitors. Therefore, PI3K inhibitors in combination with other drugs that can inhibit CSCs may be a novel attractive therapeutic strategy for the treatment of HCC.

Here, we show that PIK3C3 is an effective target against liver CSCs. PIK3C3 inhibition suppresses liver CSCs via the activation of AMPK. PIK3C3 inhibition suppress SGK3-induced liver CSCs expansion. More importantly, treatment with PIK3C3 inhibitor and PI3K inhibitor together dramatically suppresses liver CSCs expansion in vitro and in vivo, which provides a novel therapeutic intervention for HCC.

## Materials and methods

### Cell culture

The HCC cell line Huh7 was obtained from the Chinese Academy of Sciences Cell Bank (Shanghai, China). The HCC cell line MHCC-97H were obtained from the Liver Cancer Institute, Zhongshan Hospital of Fudan University (Shanghai, China). These cell lines were obtained fewer than 6 months before being used in the present study. The cell lines were authenticated using short tandem repeat markers by Genetic Testing Biotechnology Corporation (Suzhou, China). All cells were maintained in Dulbecco’s modified Eagle medium containing high glucose (Heclone), 10% fetal bovine serum (Capricorn) at 37 °C in a humidified atmosphere containing 5% CO_2_.

### Clinical HCC specimens

Liver cancer tissue microarrays of 163 patients (HLivH030PG03, HLivH060CS01, HLiv-HCC060PG-01, and HLivH180Su18) were purchased from SHANGHAI XINCHAO (Shanghai, China). HLivH180Su18 included 88 cases with 3-year follow-up information was used for survival analysis.

### siRNA transfection and chemical inhibitors

Small interfering RNAs (siRNAs) of PIK3C3 and AMPK were designed and synthesized by Guangzhou RiboBio. The sequences of the siRNAs are listed in Supplementary Table S1. Ribo FECT^™^ CP Transfection Kits (RiboBio) was used according to the manufacturer’s instruction. A total of 1–2 × 10^5^ cells were seeded and grown to 40–60% confluence per well. Transfection mixtures were prepared and added to each well. All the siRNAs and controls were used at a concentration of 100 nM. The mRNA level of target gene was detected by quantitative real-time polymerase chain reaction (qRT-PCR) and Western blot. Class I PI3K inhibitor ZSTK474, PIK3C3 inhibitors VPS34-IN-1, and VPS34-PIK-III, AICAR, metformin, rapamycin, and chloroquine were purchased from Selleck.

### Lentivirus-mediated PIK3C3 stable overexpression cells

The lentiviral vector expressing PIK3C3 and the control vector were synthesized by GENECHEM (Shanghai, China). Polybrene (GENECHEM) was used to enhance the transfection efficiency. Totally, 12–24 h before transfection, 5 × 10^4^ cells per well were seeded and grown to about 20% confluence. Then, the cells were transfected at a MOI (multiplicity of infection) of 20. After 72–96 h, the overexpression efficiency was determined by qRT-PCR and Western blot. The SGK3 stable overexpression cell lines were established in our previous study^23^.

### Spheroids formation assay

The spheroids formation assay was performed as described previously^23,24^. Briefly, single cells (5 × 10^3^/well) were seeded in serum-free culture (SFC) medium in 6-well ultra-low attachment plates (Corning). SFC medium was DMEM/F12 (Hyclone) to which was added 20 ng/ml epidermal growth factor (PeproTech), 20 ng/ml basic fibroblast growth factor (PeproTech), and 20 μl/ml B27 supplement (Life Technologies). After 1–2 weeks, the numbers of tumor spheroids (diameter > 100 μm) was counted each well.

### Western blotting

Cell lysates were extracted using RIPA buffer added with Protease Inhibitor Cocktail (Cwbio) and Phosphatase Inhibitor Cocktail (Cwbio), incubated on ice for 30 min, followed by centrifuged for 15 min at 14,000*g*. The proteins were separated by SDS-PAGE. Then proteins transferred to polyvinylidene difluoride membranes (Millipore). After being blocked with 5% defatted milk or bovine serum albumin for 1 h at room temperature, the membranes were exposed to primary antibodies overnight at 4 °C. The membranes were exposed to secondary antibodies (1:4000) at room temperature for 1 h before being revealed using enhanced chemiluminescence reagent (Engreen). The antibodies used in this study are listed in Supplementary Table S2.

### qRT-PCR

Total cellular RNAs were extracted with Trizol (Takara). The extracted RNAs were used for cDNA synthesis with the PrimeScript^™^ RT Reagent Kit (Takara) according to the manufacturer’s protocol. The cDNA was used for qRT-PCR with SYBR Premix Ex Taq (Takara) according to the manufacturer’s instruction, Ct values were detected by CFX96 Real-Time PCR Detection System (Bio-Rad). The data were analyzed using the ^2−△△Ct^ method. The primers are listed in Supplementary Table S3.

### Isolation of CD133+ cells

CD133+ cells were isolated using a CD133 MicroBead Kit (Miltenyi Biotec), performed as described previously^23^.

### Flow cytometry

Single cells (5 × 10^6^) were resuspended in 80 μl of phosphate-buffered saline (PBS) added with 20 μl FcR Blocking Reagent (Miltenyi Biotec) and 2 μl primary conjugated PE-CD133 antibody (Miltenyi Biotec) or corresponding isotype-matched control (Miltenyi Biotec), and then incubated on ice in the dark for 10 min. Then, the cells were washed 3 times with 1 ml of PBS. Then, the cells were resuspended in 500 μl of PBS and detected using a FACS Calibur (BD Biosciences).

### Immunohistochemical (IHC) staining

All slides were subjected to heat-induced epitope retrieval antigen procedure by heating in a microwave using citrate-based antigen retrieval solution. The slides were then exposed to primary antibodies at 4 °C overnight followed by incubation with biotinylated secondary antibody (DAB; Boster) at room temperature for 1 h. Reaction results were visualized by incubation with 3,3′-diaminobenzidine (DAB; Boster). The chromogenic reaction was stopped by washing with tap water, and the slides were then dehydrated in an ascending alcohol gradient, followed by cleared twice with xylene, and then mounted in neutral balsam. The percentage of positive staining areas were scored 0–4 (0, 1 [1–25%], 2 [26–50%], 3 [51–75%], and 4 [76–100%]) and staining intensity was scored 0–3 (0, negative; 1, weak; 2, moderate; and 3, strong). The overall protein expression score was calculated by multiplying the positivity and intensity scores ranging from 0 to 12. The antibodies, rabbit anti-human PIK3C3 (Abcam) and rabbit anti-human CD133 (Abcam) were used.

### In vivo xenograft experiments

All animal experiments were performed according to the Laboratory Animal Care guidelines of the Animal Ethics Committee of Chongqing Medical University. For tumorigenesis assay, MHCC97H cells were pretreated with VPS34-IN-1 (5 μM) or DMSO for 24 h. Afterward, 1 × 10^4^, 1 × 10^5^, and 1 × 10^6^ cells were injected subcutaneously in the flanks of 6-week-old female athymic nude mice. Tumor formation was observed every 3–4 days and analyzed at the eighth week. For tumor growth assay, 5 × 10^6^ MHCC97H cells were subcutaneously injected into the flanks of 6-week-old female nude mice and allowed to form tumors. Once the tumors reached about 400 mm^3^, 12 mice were randomly divided into four groups. The VPS34-IN-1 group was orally administered a dose of VPS34-IN-1 at 200 mg/kg/day for 10 days. The ZSTK474 group was orally administered a dose of ZSTK474 at 200 mg/kg/day for 10 days. The combination group was orally administered a dose of VPS34-IN-1 and ZSTK474 daily for 10 days. All inhibitors were suspended in 5% hydroxypropyl cellulose when orally administered. The control group of mice was orally administered 5% hydroxypropyl cellulose instead. Tumors were measured daily throughout the treatment period.

### Statistical analysis

All data were acquired from at least three independent experiments. SPSS 23 software were used for statistical analyses. Two independent group comparisons were analyzed using Student’s *t* test. Survival data were estimated using the Kaplan–Meier survival curves and analyzed using the log-rank test. Pearson’s correlation analysis was used to determine the correlation of PIK3C3 and CD133 expression. The results with a value of *p* < 0.05 was considered statistically significant.

## Results

### PIK3C3 is highly expressed in HCC tumors and liver CSCs

**Fig. 1:**
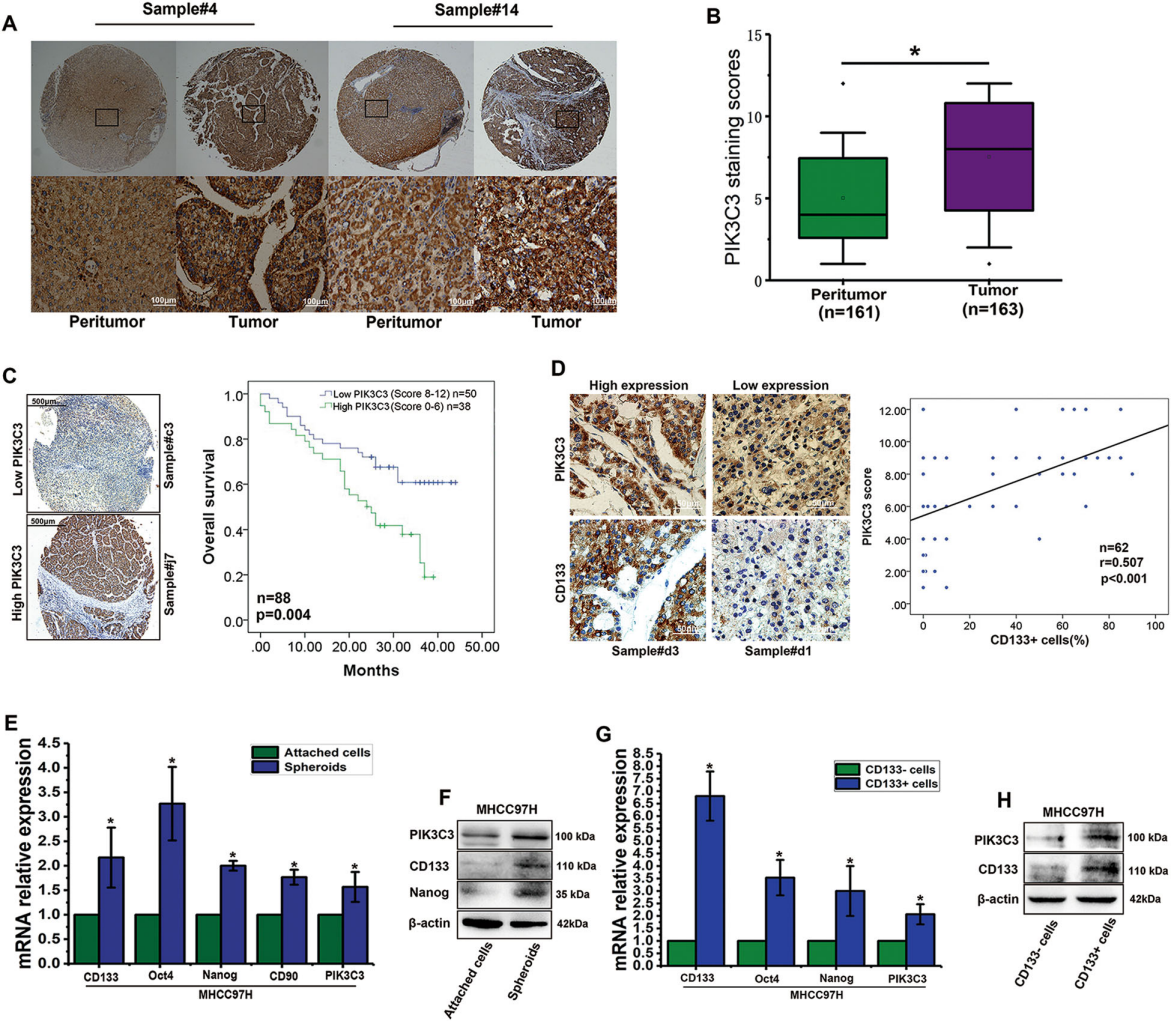
PIK3C3 is highly expressed in HCC tumors and liver CSCs. **a** IHC staining PIK3C3 images from two matched pretumor and HCC clinical samples. Scale bars, 100 μm. **b** High expression levels of PIK3C3 in HCC tumor tissues were verified by qRT-PCR. **c** Kaplan–Meier survival analysis comparing the overall survival (*n* = 88) of HCC patients with different PIK3C3 expression levels. **d** Correlation of PIK3C3 and CD133 expression in 62 HCC clinical samples. *r* = Pearson correlation coefficient. **e** The expression of liver CSC–related genes and PIK3C3 in spheroids and attached cells was compared by qRT-PCR. **f** The expression of liver CSC-related genes and PIK3C3 in spheroids and attached cells was compared by Western blot. **g** The expression of liver CSC-related genes and PIK3C3 in CD133+ and CD133 cells was compared by qRT-PCR. **h** The expression of CD133 and PIK3C3 in CD133+ and CD133 cells was compared by Western blot. All experiments were performed in triplicate, and the results are shown as mean ± standard deviation. ^*^P < 0.05.

To determine PIK3C3 expression in human HCC, IHC was conducted on commercial tissue microarrays of 163 paired tumor and peritumor tissues of HCC. We found that PIK3C3 was expressed significantly higher in HCC tumors than in the nontumor tissues (Fig. 1a, b). Kaplan–Meier analysis indicated that patients with high PIK3C3 expression in HCC tumors displayed a worse overall survival (Fig. 1c). We then analyzed available data from TCGA database. The results showed that mRNA levels of PIK3C3 in tumors were significantly higher than in nontumors (Fig. S1a), and the patients with higher PIK3C3 mRNA expression had poorer survival (Fig. S1b). Furthermore, we observed that overexpression of PIK3C3 in HCC tissues was correlated with tumor stage by analyzing clinical and pathological results in HCC samples (Supplementary Table S4). The results indicated that PIK3C3 might be a critical oncogene and play a vital role in the progression of HCC.

To further explore the relevance between PIK3C3 and liver CSCs, we first analyzed the expression correlation between PIK3C3 and liver CSCs surface marker CD133. A positive correlation between PIK3C3 and CD133 expression was revealed in a cohort of 62 HCC tumor tissues (Fig. 1d). It has been widely acknowledged that liver CSCs are highly enriched in HCC cell spheroids^25,26^. Notably, we observed that PIK3C3 was highly expressed in spheroids, which was consistent with the expression levels of several stemness related markers, including CD133, CD90, Nanog, and Oct4 (Fig. 1e, f; Fig. S1c, d). In our previous study we have isolated a subgroup of CD133+ cells from MHCC97H cells, and this CD133+ subpopulation (CSCs) possesses strong spheroids formation and tumorigenesis capacity compared with the counterpart CD133- subgroup (non-CSCs). We further confirmed that PIK3C3 was highly expressed in CD133+ cells (Fig. 1g, h). These results indicated that PIK3C3 was highly expressed in liver CSCs and HCC.

### PIK3C3 regulates expansion of liver CSCs

**Fig. 2:**
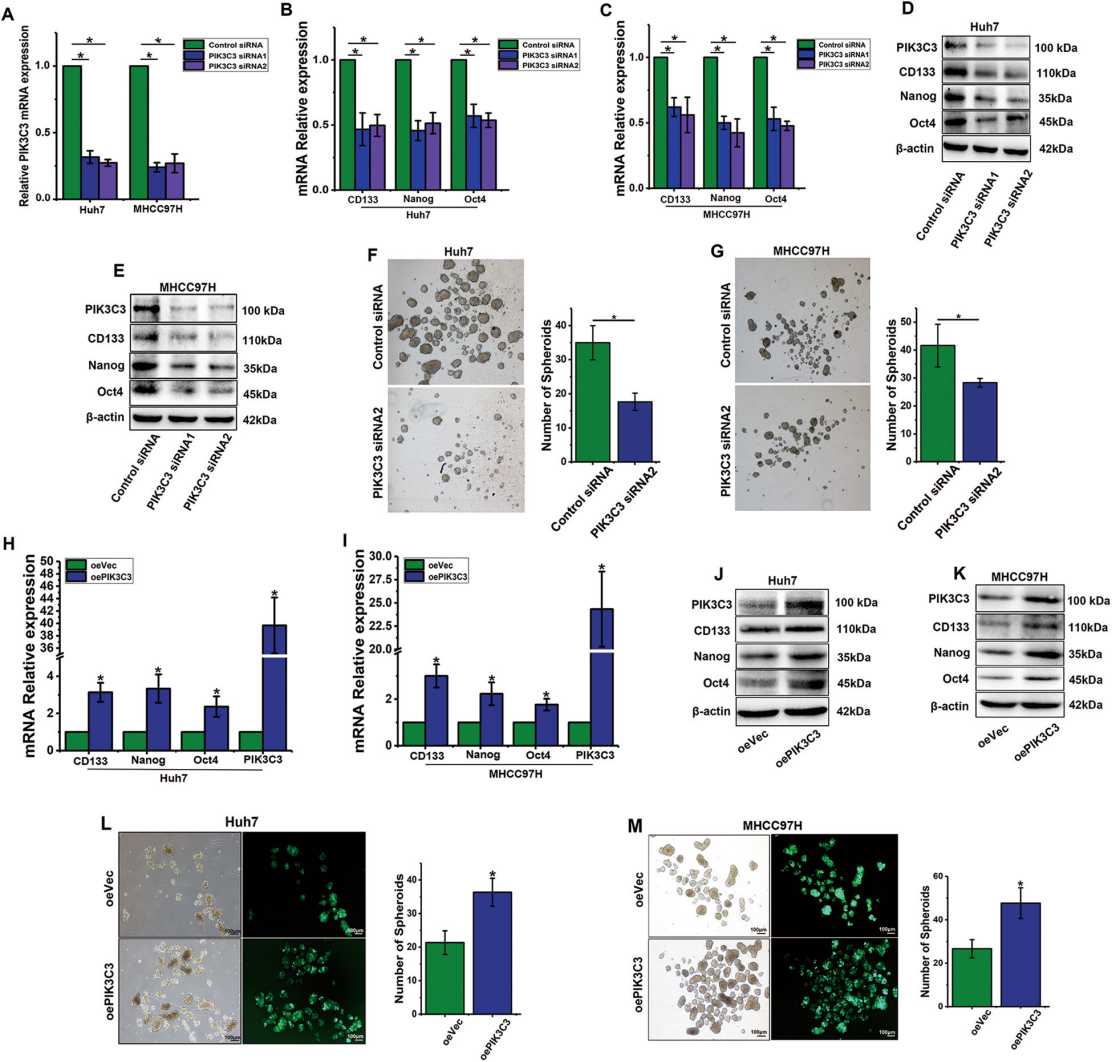
PIK3C3 regulates the expansion of liver CSCs. **a** PIK3C3 knockdown efficiency was confirmed by qRT-PCR in Huh7 and MHCC97H cells. **b**, **c** The mRNA expression levels of stemness genes after PIK3C3 knockdown in Huh7 and MHCC97H cells. **d**, **e** The protein expression levels of stemness genes after PIK3C3 knockdown in Huh7 and MHCC97H cells. **f**, **g** Spheroid formation assay of HCC cells infected by PIK3C3 siRNA2 or Control siRNA. **h**, **i** The mRNA expression levels of PIK3C3 and stemness genes in Huh7 and MHCC97H cells stably overexpressing PIK3C3 or control. **j**, **k** The protein expression levels of PIK3C3 and stemness genes in Huh7 and MHCC97H cells stably overexpressing PIK3C3 or control. **l**, **m** Spheroid formation assay of Huh7 and MHCC97H cells stably overexpressing PIK3C3 or control. All experiments were performed in triplicate, and the results are shown as mean ± standard deviation. Scale bars, 100 µm. ^*^P < 0.05.

In attempt to explore the role of PIK3C3 in liver CSC self-renewal, two siRNAs against PIK3C3 were synthesized to silence the expression of PIK3C3 (Fig. 2a). After PIK3C3 knockdown, the expression of stemness genes were significantly decreased in Huh7 and MHCC97H cells (Fig. 2b, c). The decreased expression of stemness genes, CD133, and Nanog was also determined by Western blot (Fig. 2d, e). In addition, HCC cells infected by PIK3C3 siRNA2 exhibited reduced spheroid formation compared to control cells (Fig. 2f, g). We next established a lentivirus-mediated stable PIK3C3 overexpression cell line using MHCC97H and Huh7 cells. PIK3C3 overexpression dramatically increased the expression of stemness genes (Fig. 2h, i), and the results were also determined by Western blot in protein levels (Fig. 2j, k). To confirm the effect of PIK3C3 on liver CSC self-renewal, we then conducted a spheroid formation assay to determine CSC self-renewal. Notably, PIK3C3 overexpression significantly enhanced spheroid formation (Fig. 2l, m). All together, these data indicate that PIK3C3 enhances the expansion of liver CSCs.

### PIK3C3 inhibitors repress liver CSCs in vitro and in vivo

**Fig. 3:**
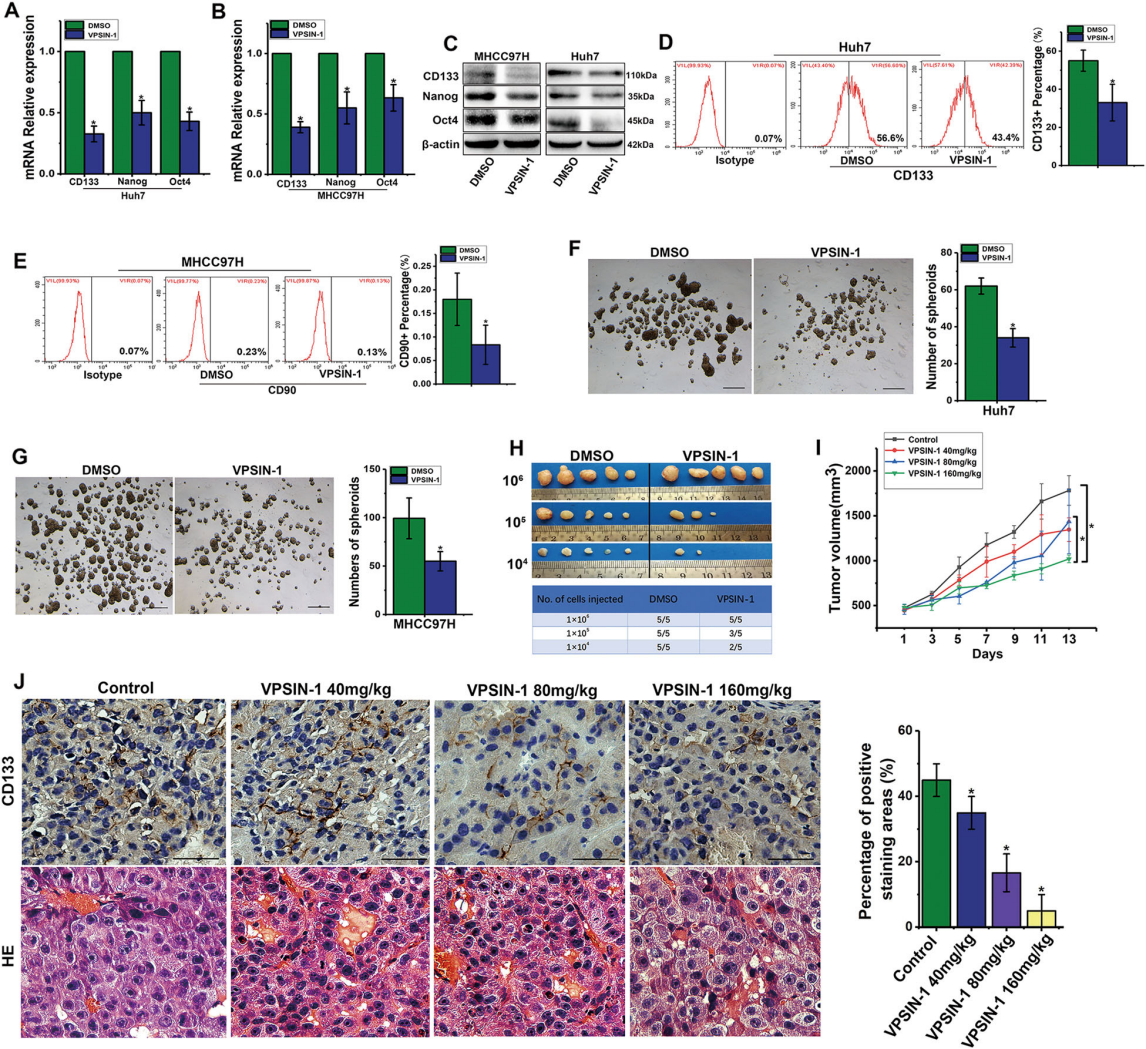
PIK3C3 inhibitors repress liver CSCs in vitro and in vivo. **a**, **b** Expression levels of stemness genes in Huh7 and MHCC97H cells treated with DMSO or VPS34-IN-1 (5 μM) detected by qRT-PCR. **c** Expression levels of stemness genes in Huh7 and MHCC97H cells treated with DMSO or VPS34-IN-1 (5 μM) detected by Western blot. **d**, **e** The proportion of CD133+ cells in Huh7 and MHCC97H cells treated with DMSO or VPS34-IN-1 (5 μM) evaluated by flow cytometric assay. **f**, **g** Spheroid formation assay of Huh7 and MHCC97H cells pretreated with DMSO or VPS34-IN-1 (5 μM). **h** Xenograft tumors derived from serial subcutaneous injections of MHCC97H cells pretreated with DMSO or VPS34-IN-1 (5 μM) for 24 h. **i** Tumor growth curves of mice bearing MHCC97H cells treated with 40, 80, and 160 mg/kg or vehicle (control). Tumor size was measured by a caliper. **j** IHC staining of CD133 in the xenografted tumors treated with VPS34-IN-1 or control. Scale bars, 50 μm. ^*^P < 0.05.

To further investigate the effect of PIK3C3 on liver CSCs, we treated HCC cells with PIK3C3 inhibitor (VPS34-IN-1) for 24 h and then detected the expression of stemness genes using qRT-PCR and Western blot. The results indicated that treated HCC cells with VPS34-IN-1 suppressed the expression of stemness genes significantly (Fig. 3a–c). Another PIK3C3 inhibitor, Vps34-PIK-III, also significantly decreased the expression of stemness genes (Fig. S2a–c). Flow cytometry analysis revealed that the percentage of CD133+ cells was decreased after MHCC97H and Huh7 cells were treated with VPS34-IN-1 for 24 h (Fig. 3d; Fig. S2d). Furthermore, CD90+ cells, another CSCs subgroup, were also inhibited by the treatment of VPS34-IN-1 (Fig. 3e). VPS34-IN-1 treatment also reduced spheroid formation (Fig. 3f, g).

To further measure the effect of inhibition of PIK3C3 on liver CSCs, a tumor initiation capacity assay was conducted in mice. We observed that VPS34-IN-1 pretreatment resulted in a significant decrease in tumor initiation ability when 10^5^ or 10^4^ cells were implanted relative to non-pretreated cells (Fig. 3h). To evaluate the effect of VPS34-IN-1 on tumor growth, we used a subcutaneous mouse xenograft model. After the tumors formed about 400 mm^3^, the mice in groups of three were orally administered 0 (control), 40, 80, and 160 mg/kg of VPS34-IN-1, respectively, for 13 days. We found that VPS34-IN-1 show significant antitumor activity in a dose-dependent manner in the treatment period as compared with control mice (Fig. 3i). IHC assay showed that tumors treated with VPS34-IN-1 decreased the percentage of CD133+ cells (Fig. 3j). All together, these data indicate that PIK3C3 plays an important role in the stemness maintenance of liver CSCs.

### PIK3C3 inhibition suppress the activation of SGK3 induced by PI3K inhibitor

**Fig. 4:**
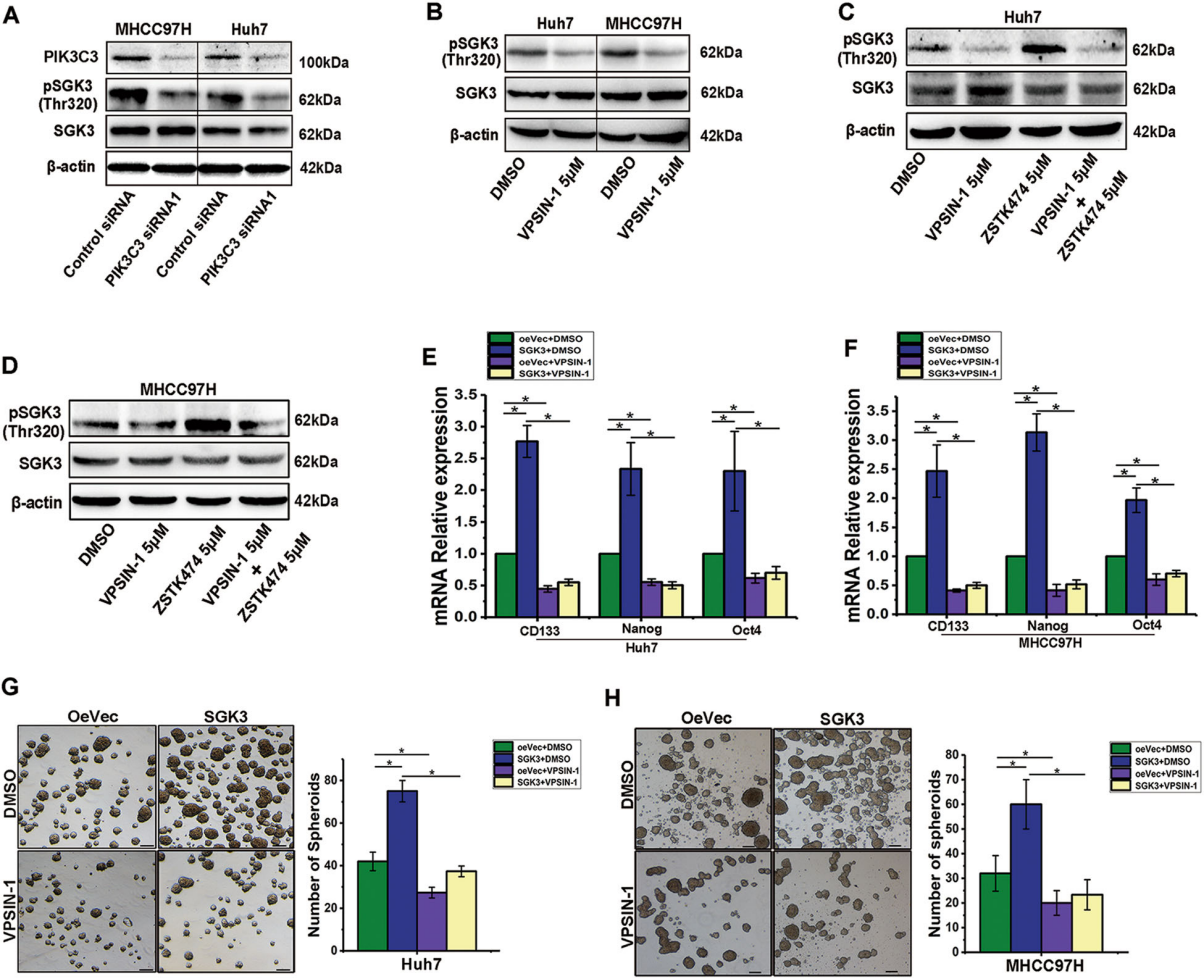
PIK3C3 inhibition suppress the activation of SGK3 induced by PI3K inhibitor. **a** Protein expression levels of pSGK3 and SGK3 in Huh7 and MHCC97H cells after PIK3C3 knockdown. **b** Protein expression levels of pSGK3 and SGK3 in Huh7 and MHCC97H cells treated with VPS34-IN-1 (5 μM). **c**, **d** Protein expression levels of pSGK3 and SGK3 in Huh7 and MHCC97H cells treated with VPS34-IN-1 (5 μM), ZSTK474 (5 μM), and both for 24 h. **e**, **f** mRNA expression levels of stemness genes in Huh7 and MHCC97H cells overexpressing SGK3 or Vector pretreated with VPS34-IN-1 (5 μM) for 24 h. Scale bars, 100 µm. **g**, **h** Spheroid formation assay of Huh7 and MHCC97H cells overexpressing SGK3 or Vector pretreated with VPS34-IN-1 (5 μM) for 24 h. Scale bars, 100 μm. ^*^P < 0.05.

Our previous study showed that prolonged inhibition of class I PI3K promotes liver CSCs expansion by activating SGK3^23^. PIK3C3 could regulate SGK3 activation by controlling PtdIns3P production via enhancing phosphorylation of T-loop and hydrophobic motifs^27^. To further explore the involvement of PIK3C3 in SGK3 activation, we therefore confirmed that PIK3C3 knock down resulted in the reduced activity of SGK3 in MHCC97H and HuH7 cells (Fig. 4a). Similarly, after PIK3C3 inhibitor treatment, we observed a significantly decrease in the expression of p-SGK3 (Fig. 4b). To investigate whether PIK3C3 inhibition could inhibit the activation of SGK3 induced by PI3K inhibitor, we treated HCC cells with PI3K inhibitor plus PIK3C3 inhibitor and assessed the phosphorylation of SGK3. We found that PIK3C3 inhibition blocked the phosphorylation of SGK3 induced by PI3K inhibitors (Fig. 4c, d). To further determine inhibition of PIK3C3 could inhibit SGK3-enhanced expansion of liver CSCs, SGK3 overexpression, and control cells were treated with VPS34-IN-1 or DMSO. VPS34-IN-1 pretreatment blocked the SGK3-enhanced expression of liver CSCs related markers (Fig. 4e, f). Similarly, spheroids formation assay showed that VPS34-IN-1 pretreatment blocked the SGK3-enhanced spheroids formation (Fig. 4c, d). The results indicated that the inhibition of PIK3C3 could block SGK3 activity induced by PI3K inhibitor and suppress liver CSC expansion induced by SGK3.

### PIK3C3 inhibition counteracts liver cancer stem cell activity induced by PI3K inhibitor

**Fig. 5:**
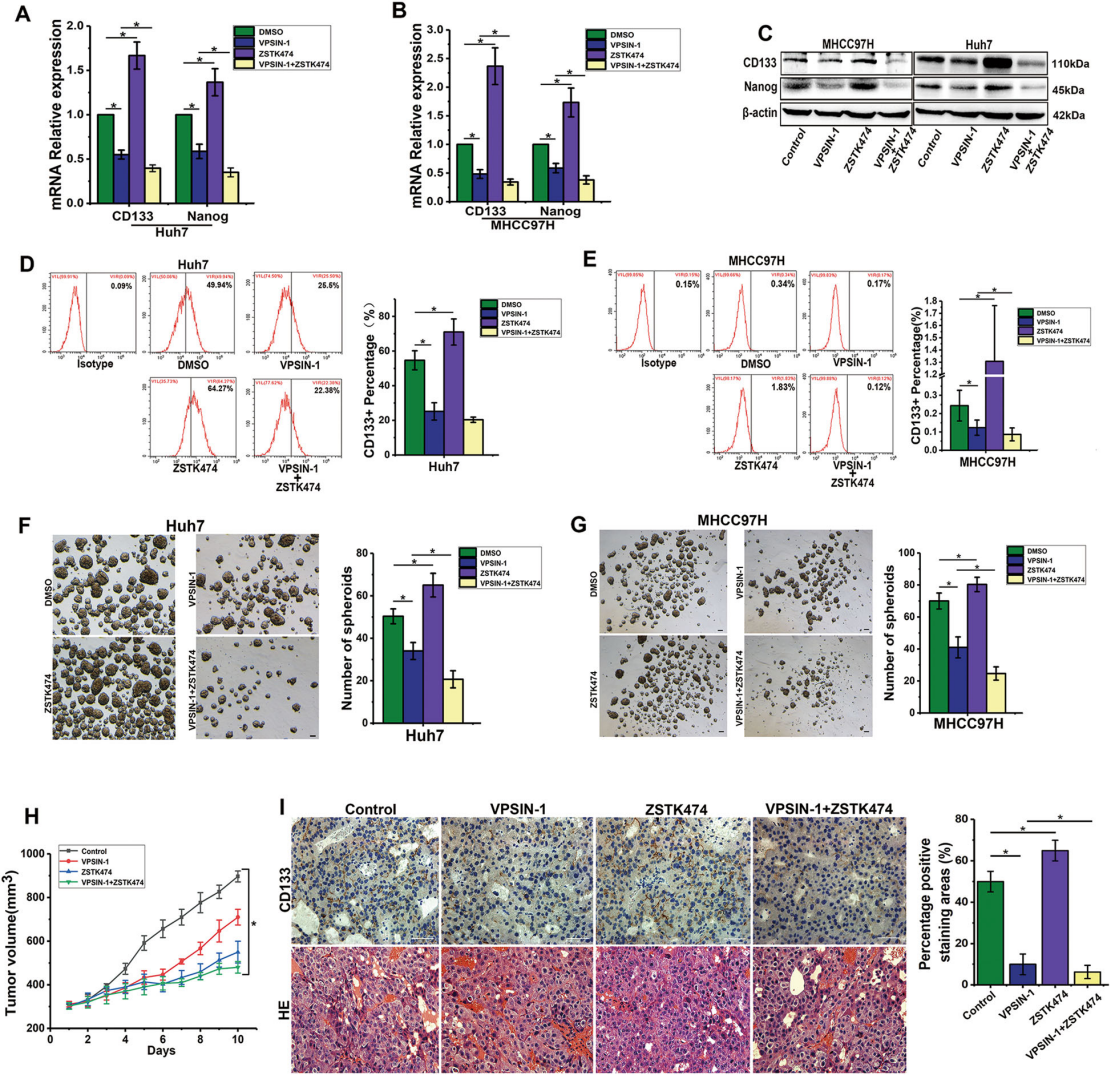
The combination of PIK3C3 and PI3K inhibitors enhances the efficacy of liver CSC inhibition. **a**, **b** mRNA expression levels of stemness genes in Huh7 and MHCC97H cells treated with VPS34-IN-1 (5 μM), ZSTK474 (5 μM), and both for 24 h. **c** Protein expression levels of stemness genes in Huh7 and MHCC97H cells treated with VPS34-IN-1 (5 μM), ZSTK474 (5 μM), and both for 24 h. **d**, **e** The proportion of CD133+ cells in Huh7 and MHCC97H cells treated with VPS34-IN-1 (5 μM), ZSTK474 (5 μM), and both for 24 h. **f**, **g** Spheroid formation assay of Huh7 and MHCC97H cells pretreated with VPS34-IN-1 (5 μM), ZSTK474 (5 μM), and both for 24 h. **h** Tumor growth curves of mice bearing MHCC97H cells treated with vehicle (control), 200 mg/kg of VPS34-IN-1, 200 mg/kg of ZSTK474, and combination of VPS34-IN-1 and ZSTK474. Tumor size was measured by a caliper. ^*^P < 0.05. **i** Immunohistochemical staining of CD133 in the xenografted tumors treated with vehicle (control), 200 mg/kg of VPS34-IN-1, 200 mg/kg of ZSTK474, and the combination of VPS34-IN-1 and ZSTK474. Scale bars, 50 µm. ^*^P < 0.05.

Since treatment of HCC cells with class I PI3K inhibitors leads to the expansion of liver CSCs via activating SGK3^23^, while PIK3C3 inhibitor could inhibit the expansion of liver CSCs via inactivating SGK3, we examined whether PIK3C3 inhibitor could abrogate the expansion of liver CSCs induced by PI3K inhibitor (ZSTK474). To our surprise, VPS34-IN-1 blocked the expansion of CSCs induced by ZSTK474; moreover, the combination of VPS34-IN-1 and ZSTK474 results in a more effective inhibitory effect on stemness genes than VPS34-IN-1 alone (Fig. 5a–c). Remarkably, cells treated with the combination of the inhibitors PIK3C3 and PI3K showed a robust elimination of CD133+ cells compared with treatment with VPS34-IN-1 alone (Fig. 5d, e). Consistently, the combination of VPS34-IN-1 and ZSTK474 results in maximal suppression of spheroid formation (Fig. 5f, g).

We further evaluated the effect of this combination strategy in vivo using a subcutaneous mouse xenograft model. Dual inhibition of PIK3C3 and PI3K resulted in a mild tumor regression in vivo compared with treatment with inhibitors alone (Fig. 5h). There was no significant difference in body weight (data not shown). Consistent with in vitro findings, we noticed decreased CD133 levels in tumors harvested from the VPS34-IN-1 treatment group compared with the control group, and the combination treatment group showed lower expression levels of CD133 than the VPS34-IN-1 treatment group, whereas ZSTK474 treatment led to an increased CD133 expression (Fig. 5i). Importantly, flow-cytometry analysis showed a significant decrease in the percentage of CD133+ cells of digested tumors in mice that had treated by VPS34-IN-1, and dual inhibition of PIK3C3 and PI3K resulted in a significant lower percentage of CD133+ cells compared with treatment with inhibitors alone (Fig. S3). Taken together, these results indicate that the dual inhibition of PIK3C3 and PI3K dramatically repressed CSC expansion in vitro and in vivo.

### PIK3C3 inhibition suppresses liver CSCs via AMPK activation

**Fig. 6:**
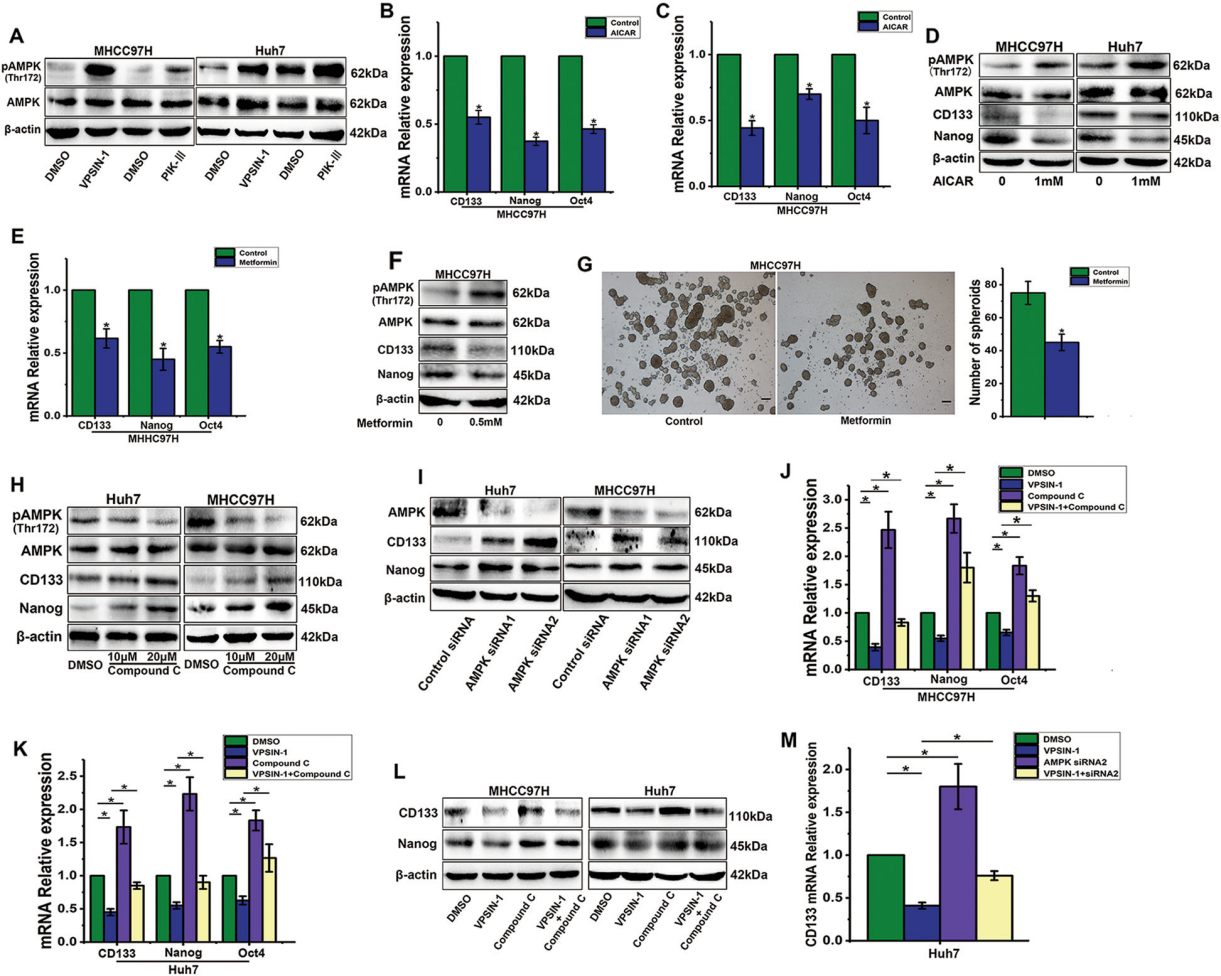
PIK3C3 inhibition suppress liver CSCs via AMPK activation. **a** Western blot analysis of AMPK total protein levels and activating phosphorylation levels in Huh7 and MHCC97H cells treated with VPS34-IN-1 (5 μM) or VPS34-PIK-III (5 μM) for 24 h. **b**, **c** Expression levels of stemness genes in Huh7 and MHCC97H cells treated with DMSO or AICAR (1 mM) for 24 h detected by qRT-PCR. **d** Expression levels of p-AMPK/AMPK and stemness genes in Huh7 and MHCC97H cells treated with DMSO or AICAR (1 mM) for 24 h detected by Western blot. **e**, **f** Expression levels of AMPK and stemness genes in Huh7 and MHCC97H cells treated with DMSO or Metformin (0.5 mM) for 24 h detected by qRT-PCR and Western blot. **g** Spheroid formation assay of MHCC97H cells pretreated with metformin (0.5 mM). **h** Expression levels of p-AMPK/AMPK and stemness genes in Huh7 and MHCC97H cells treated with DMSO or AMPK inhibitor (compound C) for 24 h detected by Western blot. **i** Expression levels of stemness genes in Huh7 and MHCC97H cells after AMPK knockdown by siRNA detected by Western blot. **j**, **k** mRNA expression levels of stemness genes in Huh7 and MHCC97H cells treated with VPS34-IN-1 (5 μM), compound C (20 mM), and both for 24 h. **l** Protein expression levels of stemness genes in Huh7 and MHCC97H cells treated with VPS34-IN-1 (5 μM), compound C (20 mM), and both for 24 h. **m** The suppression of AMPK by siRNA in MHCC-97H cells treated with 5 μM of VPS34-IN-1 resulted in a rescue of CD133 down-regulation, detected by qRT-PCR. All experiments were performed in triplicate, and the results are shown as mean ± standard deviation. Scale bars, 100 µm. ^*^P < 0.05.

VPS34 knockdown, or pharmacological inhibition of VPS34, leads to AMPK activation in confluent Caco-2 cells^11^. Upon treatment of HCC cells with VPS34-IN-1 or Vps34-PIK-III, we successfully induced AMPK activation (Fig. 6a). To examine the effect of AMPK on the self-renewal of liver CSCs, we treated HCC cells with AICAR, an activator of AMPK, and detected the expression of stemness genes using qRT-PCR and Western blot. Treatment with AICAR enhanced the activation of AMPK and decreased the expression of stemness genes significantly (Fig. 6b–d). Metformin, a first-line diabetes drug, leads to AMPK activation and is linked to CSC prevention^28^. To test the effect of metformin on AMPK activation and CSC prevention in HCC, we treated HCC cells with metformin. A significant increase in p-AMPK and decrease in expression of stemness genes was observed in HCC cells treated with metformin (Fig. 6e, f). Moreover, treatment with metformin greatly reduced spheroid formation in MHCC97H cells (Fig. 6g).

To further verify that AMPK plays an important role in regulating liver CSCs, we inhibited AMPK pharmacologically and genetically. Pharmacologic inhibition of AMPK using compound C resulted in increased stemness gene expression (Fig. 6h). Consistently, drug effects were verified by siRNA-mediated AMPK knockdown (Fig. 6i). To confirm that AMPK plays a crucial role in VPS34-IN-1–mediated CSC inhibition, we inhibited AMPK in VPS34-IN-1-treated cells and found that AMPK inhibition restored PIK3C3 repression-mediated inhibitory effects of stemness gene expression (Fig. 6j–l). Similarly, AMPK knockdown by siRNA also partially restored the expression of CD133 inhibited by VPS34-IN-1 (Fig. 6m).

### PIK3C3 regulates liver CSCs independent of the autophagy process

**Fig. 7:**
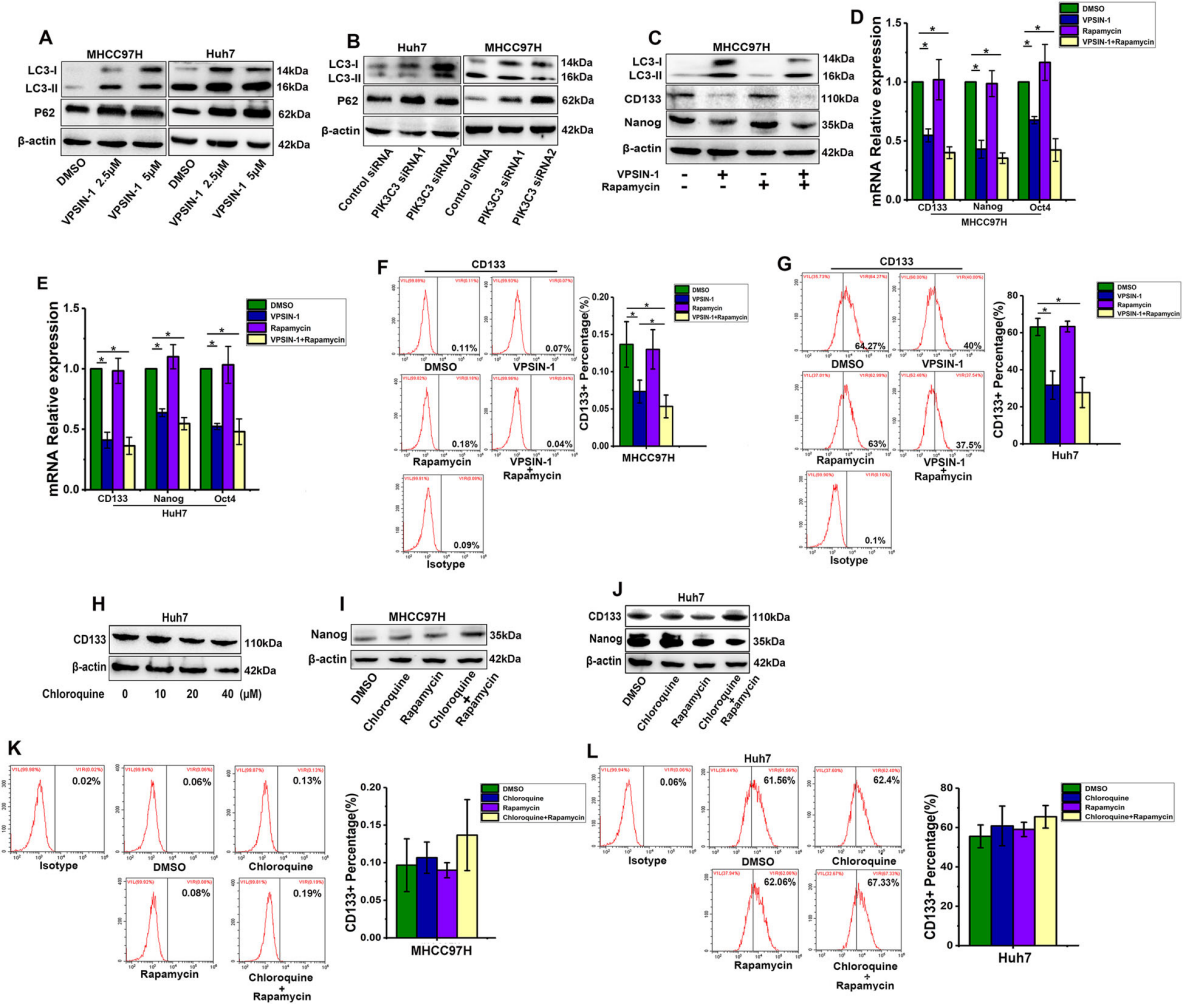
PIK3C3 regulates liver CSCs independent of the autophagy process. **a** Protein levels of LC3 and P62 were detected by Western blot in Huh7 and MHCC97H cells treated with VPS34-IN-1 (5 μM) for 24 h. **b** Protein levels of LC3 and P62 were detected by Western blot after PIK3C3 knockdown in Huh7 and MHCC97H cells. **c** Protein levels of CD133, LC3, and P62 were detected by Western blot in MHCC97H cells treated with VPS34-IN-1 (5 μM), rapamycin (200 nM), and both for 24 h. **d**, **e** mRNA levels of stemness genes were detected by qRT-PCR in Huh7 and MHCC97H cells treated with VPS34-IN-1 (5 μM), rapamycin (200 nM), and both for 24 h. **f**, **g** The proportion of CD133+ cells in Huh7 and MHCC97H cells treated with VPS34-IN-1 (5 μM), rapamycin (200 nM), and both for 24 h. **h** Expression levels of CD133 in Huh7 cells treated with chloroquine (40 μM) detected by Western blot. **i**, **j** Protein expression levels of stemness genes in Huh7 and MHCC97H cells treated with chloroquine (40 μM), rapamycin (200 nM), and both for 24 h. (**k**, **l**) The proportion of CD133+ cells in Huh7 and MHCC97H cells treated with chloroquine (40 μM), rapamycin (200 mM), and both for 24 h. All experiments were performed in triplicate, and the results are shown as mean ± standard deviation. ^*^P < 0.05.

It is well known that PIK3C3 plays a critical role in autophagy induction^29,30^. To confirm the effect of PIK3C3 on autophagy of HCC cells, we treated HCC cells with VPS34-IN-1 for 24 h and detected the expression of autophagy markers. Western blot detected a decrease in LC3-I to LC3-II conversion and accumulation of SQSTM1 (P62) in HCC cells (Fig. 7a). Consistently, LC3-I to LC3-II conversion decreased and P62 increased when PIK3C3 was silenced by siRNA (Fig. 7b). To investigate whether VPS34-IN-1 eliminates liver CSCs via the inhibition of autophagy, we treated HCC cells with VPS34-IN-1 plus rapamycin, an mTOR signaling inhibitor, to induce autophagy. Interestingly, instead of blocking the inhibited effect of VPS34-IN-1 on liver CSCs, rapamycin mildly promoted the decrease of stemness gene expression caused by VPS34-IN-1 (Fig. 7c–e). Flow cytometric analysis also showed that rapamycin could enhance the suppressive effect of VPS34-IN-1 on the proportion of CD133+ liver CSCs (Fig. 7f, g).

Cells were treated with chloroquine, a widely used autophagy inhibitor, to test whether chloroquine could inhibit liver CSCs like VPS34-IN-1. Even at the highest concentration of chloroquine treatment (40 µM), no significant decrease in CD133 expression was observed (Fig. 7h). We next treated HCC cells with chloroquine plus rapamycin to check whether rapamycin could also promote chloroquine to inhibit liver CSCs. The Western blot analysis results showed that neither chloroquine nor rapamycin treatment alone, nor the combination of the two drugs, resulted in a significant decrease in stemness genes (Fig. 7i, j). Flow cytometric analysis also showed that neither chloroquine nor rapamycin treatment alone, nor the combination of the two drugs, resulted in a significant decrease on the proportion of CD133+ liver CSCs (Fig. 7k, l). Taken together, these results indicate that PIK3C3 regulates liver CSCs independent of the autophagy process.

## Discussion

The existence of CSCs account for HCC relapse and metastasis because of their highly resistant and stem cell-like abilities^31,32^. Although many aspects of the biological behaviors and regulatory mechanisms of liver CSCs has been elucidated by accumulated evidence, strategies for their treatment are still limited. In the present study, we demonstrated that PIK3C3 plays an important role in liver CSC stemness maintenance, and inhibition of the effect of PIK3C3 by siRNA or inhibitors effectively represses liver CSCs. PIK3C3 inhibitor in combination with PI3K inhibitor exerted maximal suppression effects on liver CSCs expansion, as compared with PIK3C3 inhibitor (VPS34-IN-1) alone.

To examine whether PIK3C3 is associated with liver CSCs, we first detected the expression of PIK3C3 in HCC tissues. Our data showed that PIK3C3 was highly expressed in HCC tumors, and patients with higher PIK3C3 expression had poor survival. Moreover, we found a positive correlation between PIK3C3 and CD133 expression in a cohort of HCC tumor tissues. We have demonstrated previously that CSCs are highly enriched in HCC cell spheroids^23,24^. Our results showed a high expression level of PIK3C3 in liver CSC-enriched spheroids. Furthermore, PIK3C3 was increased in CD133+ liver CSCs compared with CD133− cells.

To further examine the role of PIK3C3 on liver CSCs, we intervened the function of PIK3C3 genetically and pharmacologically. We noted that upregulation of PIK3C3 promotes the expansion of liver CSCs, while knockdown of PIK3C3 by siRNA had opposite effects. We then treated HCC cells with PIK3C3 inhibitors, namely, VPS34-IN-1^27,33^ and Vps34-PIK-III^34^, which significantly decreased the expression of stemness genes. Furthermore, the HCC cells pretreated with VPS34-IN-1 showed a significantly lower spheroid formation and tumorigenesis abilities. In the in vivo experiments, the mice were orally administered with VPS34-IN-1 for 2 weeks. Our results showed that VPS34-IN-1 inhibited tumor growth in a dose-dependent manner and decreased CD133 expression in tumors.

The therapeutic effect targeting of the PI3K pathway has been explored by accumulated clinical studies in various cancers; however, the clinical effect was not satisfactory^21^. Our previous study reported that treatment of HCC cells with class I PI3K inhibitors leads to expansion of liver CSCs via the activation of SGK3. In the present study, we confirmed that PIK3C3 knock down or inhibition by inhibitor resulted in the reduced activity of SGK3 in HCC cells. Furthermore, PIK3C3 inhibition suppress the activation of SGK3 induced by PI3K inhibitor. We speculated that the expansion of liver CSCs may be an important factor that leads to the poor treatment outcome of PI3K inhibitors, and conjunctive therapy with PIK3C3 inhibitor may improve the antitumor effect of PI3K inhibitors. To this end, a combination of PIK3C3 inhibitor (VPS34-IN-1) and PI3K inhibitor (ZSTK474) was evaluated in in vitro and in vivo experiments. Interestingly, we found that the combination of VPS34-IN-1 and ZSTK474 results in maximal suppression of CSCs expansion of tumors in mice.

It has been reported that knockdown or pharmacological inhibition of PIK3C3 leads to the activation of AMPK^11,20^. Similarly, our data showed that inhibition of PIK3C3 by VPS34-IN-1 increased the ratio of p-AMPK to total AMPK in HCC cells. Accumulated studies supports that AMPK plays a crucial role in cancers as either a promoter^35^ or a suppressor^17^ in CSCs. To examine the effect of AMPK on liver CSC self-renewal, we treated HCC cells with AICAR and metformin, the activators of AMPK, to activate AMPK. Treatment with AICAR and metformin enhanced the activation of AMPK and significantly decreased the expression of stemness genes and spheroids formation. Furthermore, AMPK knockdown, or pharmacological inhibition of AMPK by compound C, increased the expression of stemness genes markedly. Expectedly, the inhibition or knockdown of AMPK rescued the expression of stemness genes suppressed by VPS34-IN-1.

It has been well-known that PIK3C3 plays an essential role in autophagy^29^. Our results showed that LC3-I to LC3-II conversion was decreased and P62 was increased in HCC cells treated with PIK3C3 inhibitor or siRNA, which confirmed that PIK3C3 is necessarily used in the procession of autophagy. Since autophagy has been related to a variety of CSC^36^, we speculated whether VPS34-IN-1 eliminates liver CSCs via the inhibition of autophagy. We then treated HCC cells with VPS34-IN-1 plus rapamycin to induce autophagy. Surprisingly, we observed that rapamycin did not rescue the expression of stemness genes inhibited by VPS34-IN-1. Furthermore, chloroquine or rapamycin treatment alone or the combination of the two drugs leads to no significant decrease in stemness genes in HCC cells. These results indicate that the elimination of liver CSCs by VPS34-IN-1 is independent of autophagy inhibition. We thought that autophagy may not be such an important mechanism in nutrient-rich conditions. We were surprised to find that Rapamycin could enhance the suppressive effect of PIK3C3 inhibition on liver CSCs expansion via the inhibition of mTOR, whereas the mechanism is not clear.

In conclusion, we have demonstrated that PIK3C3 plays a vital role in stemness maintenance of liver CSCs. More importantly, treatment with PIK3C3 inhibitor and PI3K inhibitor together dramatically suppress liver CSC expansion. We thought there are three main reasons for PIK3C3 inhibitor in combination with PI3K inhibitor exerted maximal suppression effects on liver CSCs expansion: (1) The inhibition of PIK3C3 could block SGK3-mediated liver CSCs expansion induced by Class I PI3K inhibitor. (2) PIK3C3 inhibition suppresses liver CSCs via AMPK activation. (3) Inhibition of mTOR could enhance the suppressive effect of PIK3C3 inhibition on liver CSCs expansion. Other interaction mechanisms may be explored in our further study. The combination of these two drugs may be an effective strategy for the treatment of liver cancer.

## Supplementary information

Supplementary Fig 1

Supplementary Fig 2

Supplementary Fig 3

Supplementary Figure Legends

Supplementary Tables
