# Supplementary figures and images for: PIK3C3 regulates the expansion of liver CSCs and PIK3C3 inhibition counteracts liver cancer stem cell activity induced by PI3K inhibitor
Source: Cell Death Dis. 2020 Jun 8;11(6):427. doi: 10.1038/s41419-020-2631-9 (PMC7280510; doi:10.1038/s41419-020-2631-9)

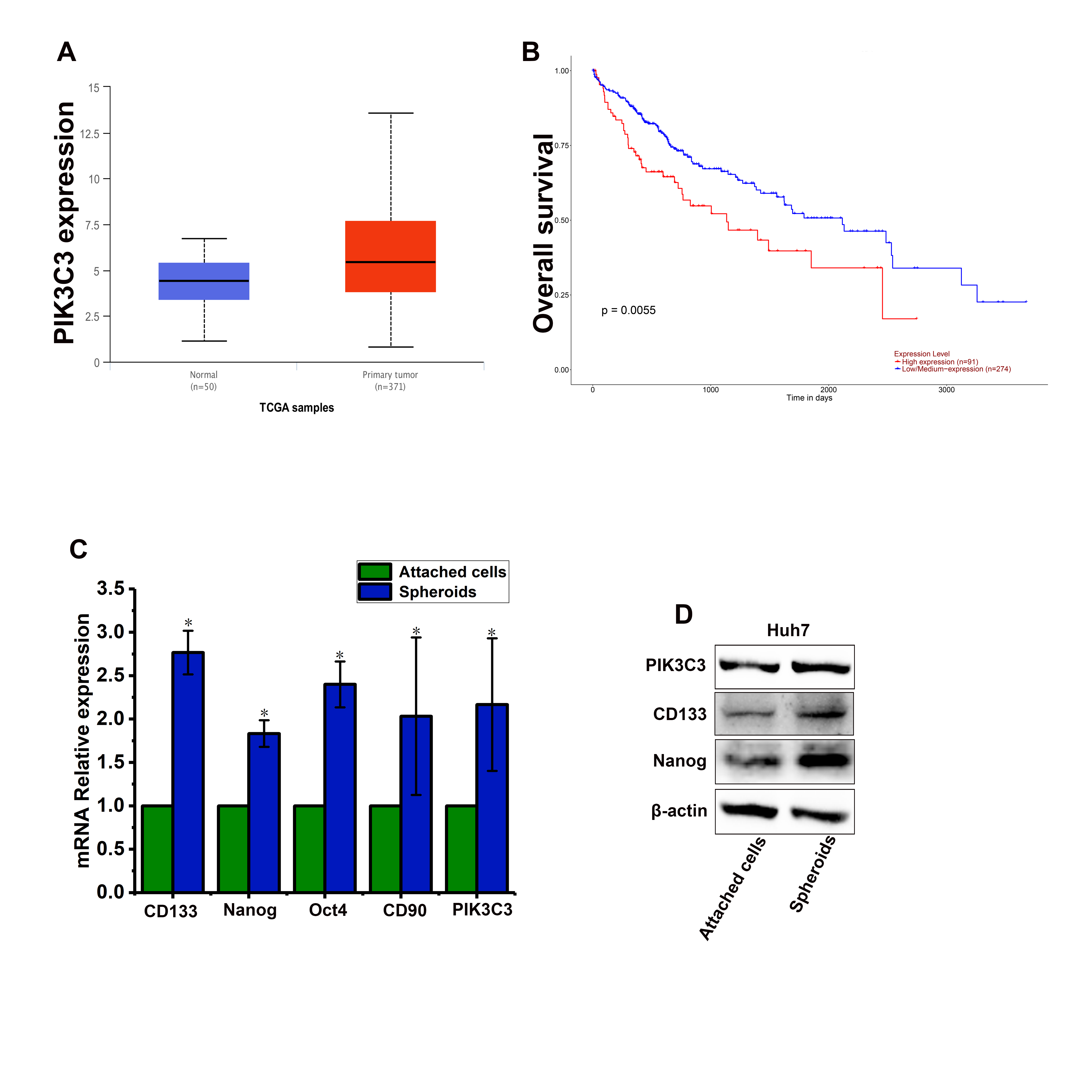

Supplement: Supplementary file 1 — Supplementary Fig 1 [file 41419_2020_2631_MOESM1_ESM.tif]

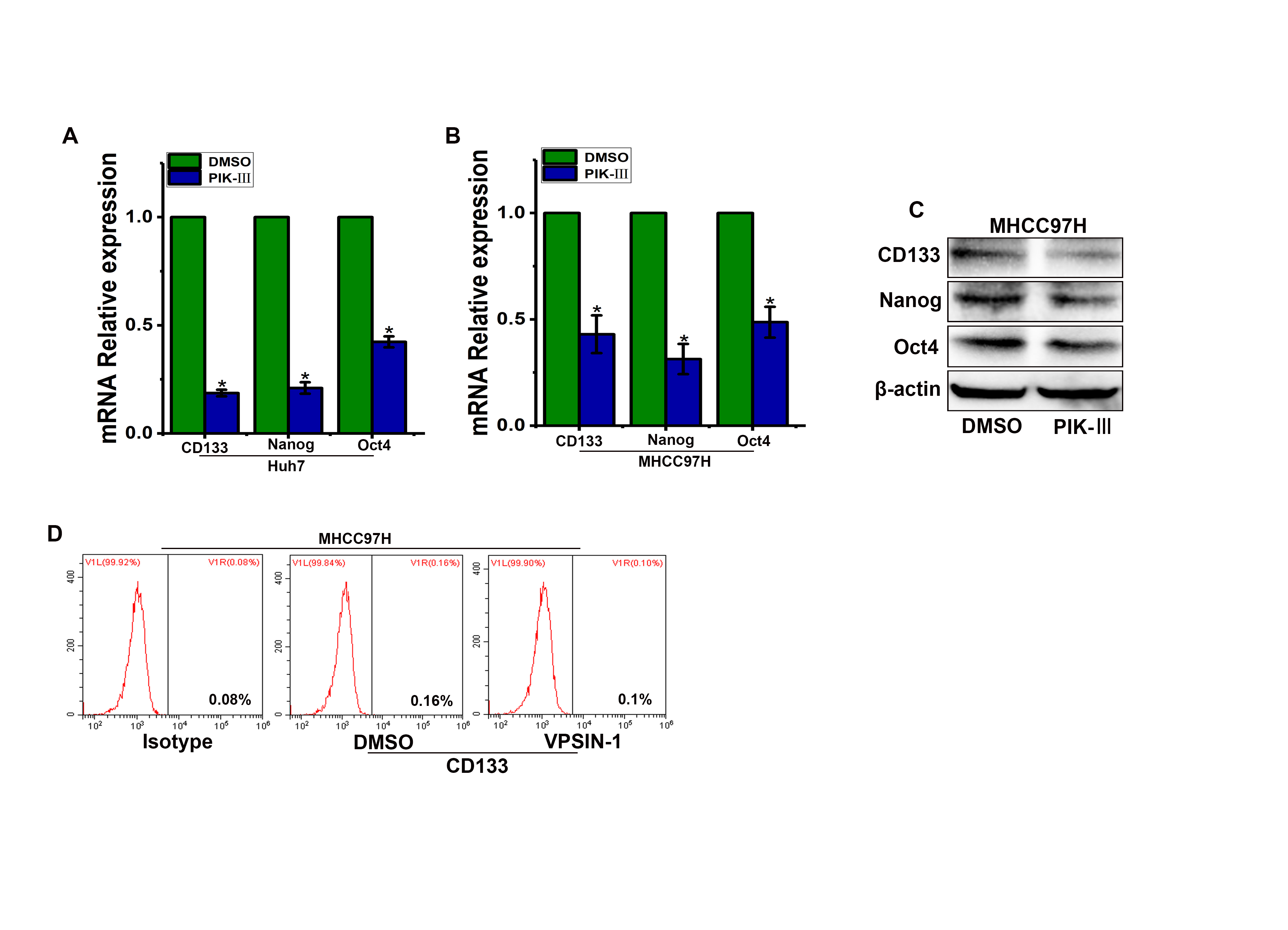

Supplement: Supplementary file 2 — Supplementary Fig 2 [file 41419_2020_2631_MOESM2_ESM.tif]

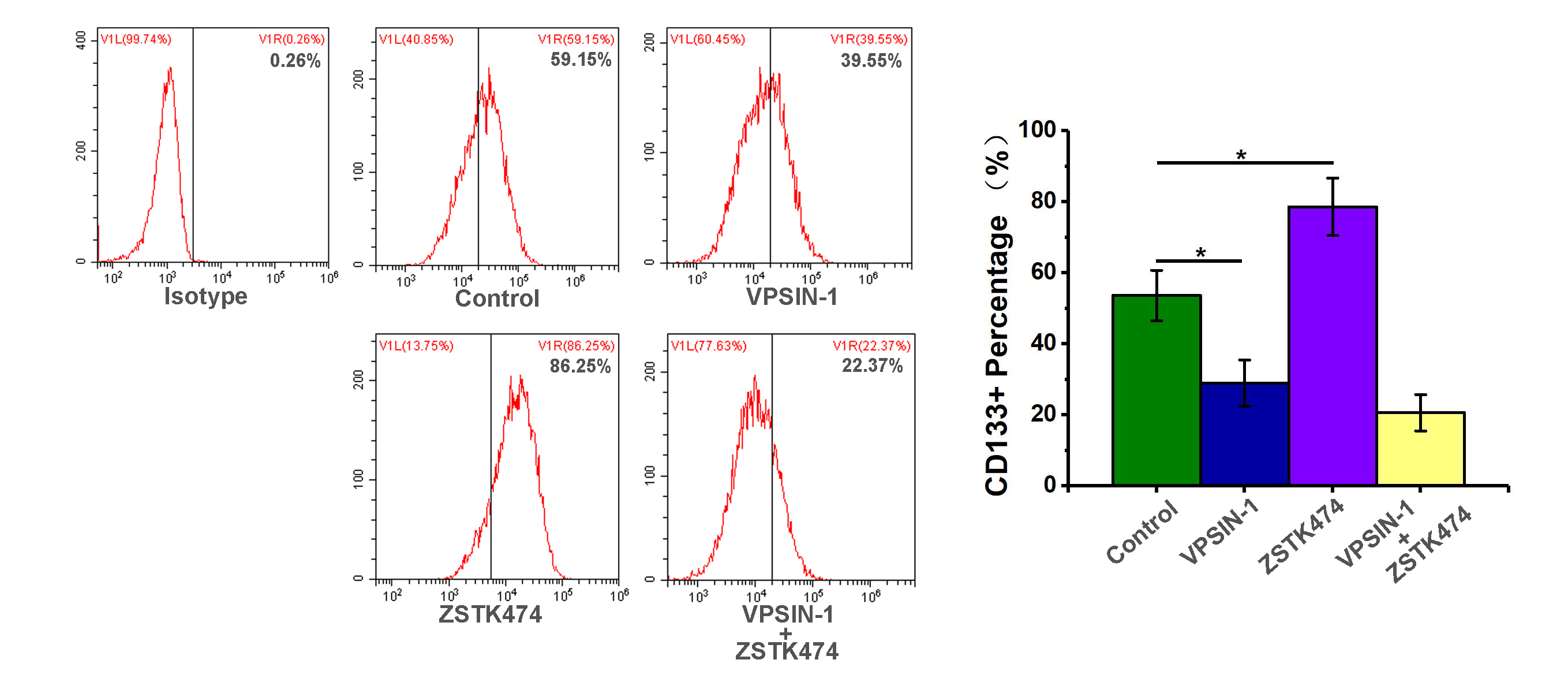

Supplement: Supplementary file 3 — Supplementary Fig 3 [file 41419_2020_2631_MOESM3_ESM.tif]
