# Supplementary material for: PIK3C3 regulates the expansion of liver CSCs and PIK3C3 inhibition counteracts liver cancer stem cell activity induced by PI3K inhibitor
Source: Cell Death Dis. 2020 Jun 8;11(6):427. doi: 10.1038/s41419-020-2631-9 (PMC7280510; doi:10.1038/s41419-020-2631-9)
Supplement: Supplementary file 4 — Supplementary Figure Legends [file 41419_2020_2631_MOESM4_ESM.docx]

**Supplementary Figure Legends**

Figure S1. PIK3C3 is highly expressed in HCC tumors and liver CSCs. (A) The mRNA expression levels of PIK3C3 in normal and tumor tissues analyzed using The Cancer Genome Atlas (TCGA) database. (B) Kaplan-Meier survival analysis comparing the overall survival of HCC patients with different PIK3C3 expression levels analyzed by TCGA database. (C, D) The expression of liver CSC-related genes and PIK3C3 in spheroids and attached cells was compared by Western blot in Huh7 cells. *p＜0.05.

Figure S2. PIK3C3 inhibitors repress liver CSCs self-renewal in vitro. (A, B) Expression levels of stemness genes in Huh7 and MHCC97H cells treated with DMSO or VPS34-PIK-III detected by qRT-PCR. (C) Expression levels of stemness genes in MHCC97H cells treated with DMSO or VPS34-PIK-III detected by Western blot. (D) The proportion of CD133+ cells in MHCC97H cells treated with DMSO or VPS34-PIK-III evaluated by flow cytometric assay. *p＜0.05.

Figure S3. flow-cytometry analysis of CD133 cell surface expression in cells isolated from tumors of mice treated with vehicle (control), 200 mg/kg of VPS34-IN-1, 200 mg/kg of ZSTK474, and the combination of VPS34-IN-1 and ZSTK474. *p＜0.05.
