# Supplementary material for: PIK3C3 regulates the expansion of liver CSCs and PIK3C3 inhibition counteracts liver cancer stem cell activity induced by PI3K inhibitor
Source: Cell Death Dis. 2020 Jun 8;11(6):427. doi: 10.1038/s41419-020-2631-9 (PMC7280510; doi:10.1038/s41419-020-2631-9)
Supplement: Supplementary file 5 — Supplementary Tables [file 41419_2020_2631_MOESM5_ESM.docx]

**Supplementary Tables**

Supplementary Table S1

Sequences of the small siRNA

| Gene |
| --- |
| PIK3C3 siRNA1 ACTCCTGGCAGAACAAGTA |
| PIK3C3 siRNA2 CTACCAAGAAGGATAGTCA |
| AMPK siRNA1 CCUCAAGCUUUUCAGGCA |
| AMPK siRNA2 CAAAGUCGACCAAAUGAUA |

Supplementary Table S2

Antibodies used in this study

| Target Protein | Vendor | Catalog NO. |
| --- | --- | --- |
| PIK3C3 | Abcam | ab124905 |
| CD133 | Abcam | ab226355 |
| Oct4 | Immunoway | YM1220 |
| Nanog | Immunoway | YM0464 |
| AMPK | CST | 2795 |
| Phospho-AMPKα (Thr172) | CST | 2535T |
| DRP1 | Immunoway | YT1414 |
| Phospho-DRP1 (Ser616) | CST | 4494 |
| DRP1 (phospho Ser637) | Immunoway | YP0841 |
| LC3 | CST | 3868 |
| P62 | CST | ab109012 |
| β-Actin | Immunoway | YM0012 |

Supplementary Table S3

Primers used in this study

| Genes | Forward primer (5’-3’) | Reverse primer (5’-3’) |
| --- | --- | --- |
| PIK3C3 | CTGTGAGGCGTTATGCTGTT | GGTAGGTTCCAATCCATTCTTT |
| Nanog | AATACCTCAGCCTCCAGCAGATG | TGCGTCACACCATTGCTATTCTTC |
| Oct4 | CTTGCTGCAGAAGTGGGTGGAGGAA | CTGCAGTGTGGGTTTCGGGCA |
| CD90 | GACCCGTGAGACAAAGAAGC | GCCCTCACACTTGACCAGTT |
| CD133 | TGGATGCAGACCTTGACAACGT | ATACCTGCTACGACAGTCGTGGT |
| β-Actin | CCTGGCACCCAGCACAAT | GGGCCGGACTCGTCATAC |

Supplementary Table S4

Correlation of endogenous PIK3C3 expression and clinicopathological features in HCC

| Variable | PIK3C3 expression  Low High | | p value |
| --- | --- | --- | --- |
| Gender  Male  Female | 61  16 | 72  13 | 0.415 |
| Age (years)  ＜ 50  ≥ 50 | 28  40 | 23  61 | 0.085 |
| Tumor size  ＜ 5 cm  ≥ 5 cm | 24  50 | 24  59 | 0.729 |
| Tumor stage  Ⅰ  Ⅱ  Ⅲ | 16  32  21 | 8  50  32 | *0.045 |

* p<0.05
